# From Composition to Shelf Life: Oxidative Deterioration Trajectories of Monovarietal Extra Virgin Olive Oils

**DOI:** 10.3390/plants15182834

**Published:** 2026-09-16

**Authors:** Cosimo Taiti, Giovanni Spinelli, Elettra Marone, Elisa Masi, Stefano Mancuso

**Affiliations:** 1 Department of Agriculture, Food, Environment and Forestry (DAGRI), University of Florence, Viale Delle Idee 30, Sesto F. No, 50019 Florence, Italy; giovanni.spinelli@unifi.it (G.S.); elisa.masi@unifi.it (E.M.); stefano.mancuso@unifi.it (S.M.); 2 Department of Bioscience and Technology for Food, Agriculture and Environment, University of Teramo, Via Renato Balzarini 1, 64100 Teramo, Italy; emarone@unite.it; 3 Fondazione per il Futuro delle Città, Via Boccaccio 50, 50133 Firenze, Italy

**Keywords:** olive cultivars, EVOO shelf life, oxidative defect, MUFA:PUFA ratio, volatile fingerprint

## Abstract

The commercial shelf life of extra virgin olive oil (EVOO) is commonly expressed as a fixed best-before period, although its actual duration may differ considerably among oils. This study investigated the chemical, volatile and sensory evolution of six filtered monovarietal Italian EVOOs during 16 months of controlled storage to identify initial compositional factors associated with cultivar-dependent shelf life. Oils were bottled in dark glass and stored at 15 ± 1 °C. Initial chemical composition was determined, while volatile fingerprints by PTR-ToF-MS and IOC sensory characteristics were monitored every four months. All oils initially fulfilled IOC requirements for the extra virgin category but followed distinct deterioration trajectories. Frantoio (Siena) and Intosso maintained the extra virgin category for at least 12 months, whereas Frantoio (San Piero a Sieve), Gentile di Chieti, Itrana and Leccino developed oxidation defects within this period. Sensory deterioration was associated with progressive depletion of lipoxygenase-derived volatile compounds and accumulation of oxidation-related VOCs. Among the investigated compositional variables, the MUFA:PUFA ratio was most consistently associated with shelf-life behaviour. These findings indicate that EVOO shelf life reflects cultivar-dependent oxidative deterioration trajectories largely associated with initial oil composition.

## 1. Introduction

Extra virgin olive oil (EVOO) is widely recognized as one of the highest-quality edible vegetable oils because of its distinctive sensory characteristics, high nutritional value, and well-documented health-promoting properties. Preserving these attributes throughout storage and commercialization is therefore essential to ensure compliance with the declared commercial category and to protect both consumers and producers. However, EVOO is not a chemically inert product. Oxidative and hydrolytic reactions begin immediately after extraction and progressively modify its physicochemical, volatile and sensory characteristics throughout storage [1,2]. The rate of these degradative processes depends on both extrinsic (storage and packaging conditions) and intrinsic (initial chemical composition) factors. Storage temperature, oxygen availability, light exposure, packaging material and storage duration all influence the evolution of olive oil quality, whereas the intrinsic susceptibility of the oil to oxidative deterioration is largely determined by its initial chemical composition, particularly its fatty acid profile and endogenous antioxidant content [3,4]. Filtration is widely adopted immediately after extraction to improve storage stability by removing suspended solids, residual vegetation water, enzymes, microorganisms and trace metals that promote hydrolytic and fermentative degradation [5]. Although filtration may influence some minor bioactive constituents [6], it is generally recognized as one of the most effective technological practices for limiting microbial activity and improving the microbiological and oxidative stability of EVOO during storage [7]. Nevertheless, filtration cannot prevent the progressive autoxidation of the lipid fraction, and even properly produced and correctly stored oils inevitably undergo quality deterioration over time [8]. During storage, oxidative reactions progressively modify the chemical composition of EVOO through the depletion of endogenous antioxidants and desirable volatile compounds together with the accumulation of oxidation products [2,9,10]. As deterioration progresses, positive sensory attributes decline and oxidation defects may eventually become perceptible, leading to the loss of the extra virgin category even when the principal physicochemical quality parameters still comply with the legal limits established for EVOO. According to the regulatory criteria established by the International Olive Council (IOC), classification as extra virgin olive oil requires compliance with specific physicochemical parameters and sensory requirements, including the absence of perceptible sensory defects and the presence of fruity attributes. Consequently, the period during which an olive oil remains marketable as extra virgin olive oil does not necessarily coincide with the period during which it remains chemically compliant with regulatory standards. This discrepancy has been highlighted by several market surveys. Frankel et al. [11], Casadei et al. [12] and Taiti et al. [13] reported that a substantial proportion of commercial olive oils marketed as “extra virgin” no longer fulfilled the sensory requirements established by IOC. These findings indicate that compliance at bottling does not necessarily guarantee maintenance of the extra virgin category throughout the commercial life of the product. Shelf life is generally defined as the period during which a food product maintains its safety and acceptable quality under specified storage conditions [14]. For EVOO, however, the relevant endpoint is not simply how long the oil remains chemically stable or suitable for consumption, but rather how long it preserves the physicochemical and sensory characteristics required for classification as extra virgin olive oil. Although the “best before” date reported on commercial labels is commonly established between 18 and 24 months according to producers’ experience and current industry practice, it remains unclear whether this period actually corresponds to the time during which bottled EVOOs retain the characteristics required for the extra virgin category. Since the “best before” date is determined by the food business operator rather than being established by legislation, its scientific reliability ultimately depends on the availability of experimental evidence capable of supporting realistic shelf-life estimations. Furthermore, EVOOs differ substantially in their intrinsic susceptibility to oxidative deterioration because of differences in their initial chemical composition, particularly the balance between fatty acid composition and endogenous antioxidants. This compositional variability reflects the combined effects of genotype and growing environment [4]. Consequently, it is unlikely that a single storage period can adequately describe the commercial shelf life of all extra virgin olive oils. However, the initial compositional characteristics most closely associated with these different deterioration rates remain poorly understood. Several studies have extensively investigated the effects of storage conditions, packaging systems and filtration on EVOO stability, showing that increased temperature, oxygen availability and light exposure generally accelerate oxidative deterioration, whereas storage conditions and packaging systems limiting these factors contribute to preserving chemical and sensory quality [1,3,7,9,10]. More recently, Proton Transfer Reaction–Time of Flight–Mass Spectrometry (PTR-ToF-MS) has proven effective in discriminating high-quality EVOOs from oils affected by sensory defects and in monitoring aroma evolution during olive oil processing and storage [13,15]. However, considerably less is known about how the initial chemical composition of bottled EVOOs influences the subsequent evolution of their volatile profile and, ultimately, the duration of the extra virgin category during storage. In particular, it remains unclear which initial compositional characteristics are most closely associated with the different deterioration rates observed among oils stored under identical conditions.

Therefore, the present study addressed two complementary questions. First, for how long do bottled monovarietal extra virgin olive oils retain the sensory characteristics required for classification as extra virgin olive oil under controlled storage conditions? Second, which initial compositional characteristics are most closely associated with the different deterioration rates observed among oils? To answer these questions, the evolution of six filtered monovarietal EVOOs was monitored over 16 months by integrating IOC sensory evaluation with PTR-ToF-MS volatile fingerprinting, while the initial chemical composition of each oil was investigated to identify the parameters most closely associated with its subsequent shelf-life behaviour.

## 2. Results and Discussion

### 2.1. Chemical Analysis

All analytical parameters complied with the legal limits established for the extra virgin olive oil category (Table 1). Moreover, all oils exhibited chemical characteristics consistent with fresh, high-quality extra virgin olive oils, as indicated by their very low free acidity (0.13–0.27%), low peroxide values (3.73–7.60 mEq O_2_ kg^−1^), high oleic acid content (65.6–74.4%), and moderate to high total phenolic concentrations (286–709 mg kg^−1^). Free acidity showed only limited variability among samples, ranging from 0.13 ± 0.01% in Gentile di Chieti and Itrana oils to 0.27 ± 0.03% in Moraiolo oil, with all values remaining well below the legal limit of 0.8% for extra virgin olive oil. These consistently low values reflect the use of healthy olives and appropriate harvesting and processing practices. In contrast, significant differences were observed for peroxide value and total phenolic content. The highest peroxide value was recorded in the Frantoio oil produced in Siena (7.00 ± 0.51 mEq O_2_ kg^−1^), whereas the lowest was found in Peranzana oil (3.73 ± 0.81 mEq O_2_ kg^−1^), although all values remained well below the regulatory limit of 20 mEq O_2_ kg^−1^, indicating excellent oxidative status. Likewise, total phenolic content differed significantly among the olive oils analysed, ranging from 286.33 ± 16.01 mg kg^−1^ in Gentile di Chieti to 709.33 ± 28.10 mg kg^−1^ in Moraiolo. The relatively high phenolic contents observed are likely attributable, at least in part, to the early harvesting stage adopted for all cultivars, which is known to preserve phenolic compounds by limiting the progressive decline associated with fruit ripening [16]. Beyond confirming the high initial quality of all EVOOs, the chemical characterization revealed marked differences in the compositional variables known to influence oxidative stability during storage (Table 1). As reported by Emmanouilidou and Kyriacou [17], oleic, linoleic, and linolenic acids are the principal fatty acids in virgin olive oils, owing to their nutritional importance and their key influence on oxidative stability. Their relative proportions are largely determined by genotype, although environmental conditions and their interaction may substantially modulate the final fatty acid profile by influencing fatty acid desaturation during fruit development [17]. Consequently, the balance between monounsaturated and polyunsaturated fatty acids reflects the combined influence of genotype and growing environment and represents one of the principal compositional determinants of oxidative stability in virgin olive oils. In all cultivars, oleic acid was the predominant fatty acid, followed by palmitic and linoleic acids, in agreement with the characteristic composition of virgin olive oil. Most fatty acids differed significantly among cultivars, consistent with previous reports [18,19]. Both genetic and environmental factors contributed to the observed differences. For example, oleic acid content ranged from 68.57 ± 0.74% in Gentile di Chieti to 74.36 ± 0.60% in Intosso. Although both cultivars are native to the Abruzzo region and were grown in the same orchard under identical agronomic conditions, they exhibited markedly different oleic acid contents, highlighting the predominant influence of genotype. Conversely, the two Frantoio oils, representing the same genotype grown at two different sites, displayed comparable oleic acid concentrations but differed significantly in linoleic and linolenic acid contents. Since harvest date and malaxation time were identical and fruit maturity was similar between the two samples, these differences are consistent with site-associated variation in fatty acid composition. However, the present experimental design does not allow the contribution of individual environmental or orchard-related factors to be disentangled, nor was fatty acid desaturase activity directly measured. Therefore, the observed differences should not be interpreted as direct evidence of environmentally induced modulation of desaturase activity, although previous studies have shown that temperature and other pedoclimatic factors can influence fatty acid desaturation and the resulting fatty acid profile in olive [17,18,20,21]. More broadly, the interplay between genetic background and environmental temperature in shaping quality-related traits is increasingly recognized across crop species. For example, Li et al. [22] recently demonstrated in rice that natural genetic variation in a temperature-responsive regulatory system results in contrasting grain-quality outcomes under high-temperature field conditions, further highlighting the relevance of genotype-by-environment interactions in determining crop quality.

Particular attention was paid to the relative abundance of monounsaturated fatty acids (MUFAs) and polyunsaturated fatty acids (PUFAs = linoleic + linolenic acids), as well as to the MUFA:PUFA ratio, since these parameters are closely associated with the oxidative stability of virgin olive oils. Because polyunsaturated fatty acids are considerably more susceptible to oxidation than oleic acid, the MUFA:PUFA ratio is widely recognized as one of the most reliable indicators of the intrinsic oxidative stability of virgin olive oils [23,24]. Total MUFA content ranged from 69.67% in Gentile di Chieti to 75.27% in Intosso, whereas total PUFA content ranged from 7.90% in Frantoio (Siena) to 11.52% in Gentile di Chieti. Notably, the two Frantoio oils also differed in PUFA content, with values of 7.90% (Siena) and 9.88% (San Piero a Sieve), highlighting site-associated variation in fatty acid composition within the same cultivar. Accordingly, the MUFA:PUFA ratio ranged from 6.05 in Gentile di Chieti to 9.40 in Frantoio produced in Siena. Among the cultivars analysed, Frantoio produced in Siena (9.40), Intosso (9.07) and Leccino (8.09) exhibited the highest ratios, whereas Gentile di Chieti (6.05) showed the lowest value, reflecting its comparatively high PUFA content. The difference observed between the two Frantoio oils (9.40 in Siena and 7.33 in San Piero a Sieve) further highlights site-associated variation in fatty acid composition and, consequently, in the potential oxidative stability of the oils. Since fatty acid composition is a major determinant of oxidative stability, the observed differences in MUFA:PUFA ratio indicate that the investigated oils possessed distinct intrinsic susceptibilities to autoxidation during storage. Higher MUFA:PUFA ratios are generally associated with greater resistance to autoxidation, although the actual stability of virgin olive oil also depends on phenolic compounds and other endogenous antioxidants [25]. Overall, although all oils initially fulfilled the chemical requirements for the extra virgin category and exhibited characteristics consistent with high-quality EVOOs, their markedly different fatty acid composition and phenolic content suggested distinct intrinsic susceptibilities to oxidative deterioration. The subsequent sensory and volatile monitoring was therefore undertaken to investigate whether the observed differences in initial chemical composition were associated with different oxidative trajectories during storage and, ultimately, with the maintenance of the extra virgin category.

### 2.2. Sensory Evaluation

**Figure 1:**
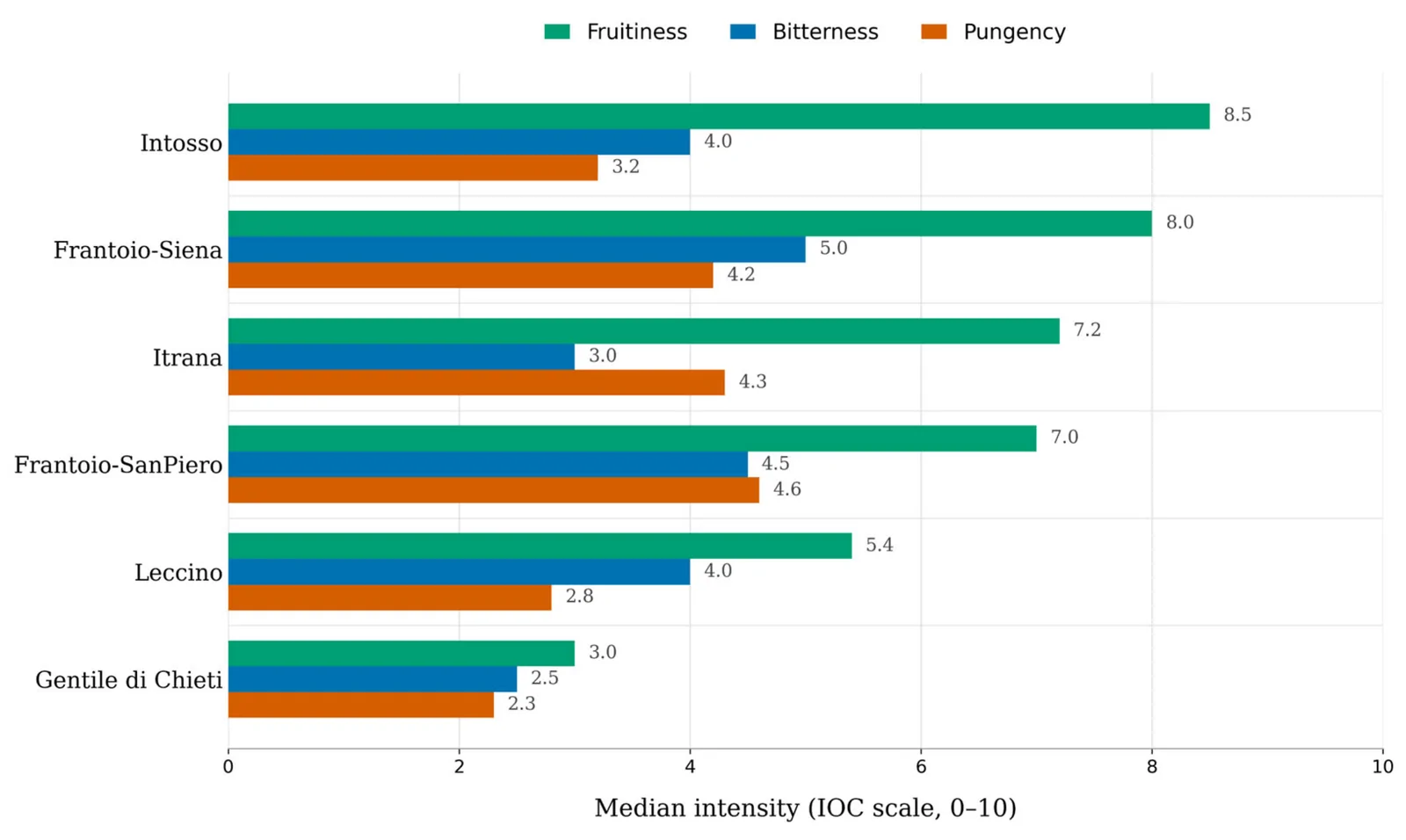
Sensory profile of the investigated extra virgin olive oils at bottling (T0). Bars indicate the median intensity of the positive sensory attributes (fruitiness, bitterness and pungency) determined according to the IOC official panel test method. No sensory defects were detected in any sample (median of defect = 0), and all oils complied with the IOC criteria for classification as extra virgin olive oil.

The results of the sensory evaluation during storage are summarized in Table 2. At bottling (T0), all olive oils fulfilled the IOC sensory requirements for classification as EVOO, being free of sensory defects while exhibiting cultivar-dependent differences in the intensity of positive sensory attributes (Table 2; Figure 1). However, clear cultivar-dependent differences emerged during storage in both the rate of fruitiness decline and the onset of oxidation defects. Fruitiness ranged from 3.0 in Gentile di Chieti to 8.5 in Intosso, whereas bitterness and pungency also showed cultivar-dependent differences, highlighting the distinctive sensory profiles of the investigated oils (Figure 1). The wide variability observed among cultivars at T0 is consistent with previous studies reporting that the sensory characteristics of extra virgin olive oils are strongly influenced by both cultivar and geographical origin [26,27,28]. From the subsequent sampling times (T1–T4), the sensory evaluation focused exclusively on perceived defects and fruitiness, since the absence of sensory defects together with the presence of fruitiness are the sensory criteria required for IOC classification. After 4 months of storage (T1), all oils remained free of sensory defects, although a slight decline in fruitiness was already evident in most cultivars. Gentile di Chieti exhibited the fastest sensory deterioration, with fruitiness decreasing progressively from 3.0 at T0 to 1.8 after 8 months of storage (T2), despite the absence of detectable sensory defects at this stage. Therefore, no oxidation defects were detected in any cultivar during the first 8 months of storage, although cultivar-dependent reductions in fruitiness were already evident. After 12 months of storage (T3), this cultivar exhibited the highest oxidation defect intensity (2.0), together with the lowest fruitiness score (1.1). Oxidation-related defects also became perceptible in Frantoio produced in San Piero a Sieve (1.2), Itrana (1.6) and Leccino (1.5), indicating that these oils no longer complied with the IOC sensory requirements for the extra virgin olive oil category. In contrast, Frantoio produced in Siena and Intosso maintained the absence of sensory defects throughout the first 12 months of storage while preserving comparatively higher fruitiness scores (4.0 and 4.2, respectively). These two cultivars exhibited the greatest sensory stability during storage, with oxidation defects becoming perceptible only after 16 months (T4), when defect intensities reached 1.2 and 1.0, respectively. Consequently, at this stage, none of the investigated oils could still be classified as extra virgin olive oil according to IOC standards. A common trend observed across all cultivars was that fruitiness declined progressively before oxidation defects became perceptible, indicating that sensory deterioration began well before the oils no longer fulfilled the IOC criteria for the extra virgin olive oil category. This behaviour is consistent with the observations of Lobo-Prieto et al. [29], who reported that the progressive decline of fruitiness preceded the appearance of oxidation defects during EVOO storage. Interestingly, the oils exhibiting the longest sensory shelf life also displayed among the highest initial fruitiness intensities. However, whether this association reflected a direct effect of the initial sensory profile or rather differences in oxidative stability could only be clarified by integrating the sensory results with the initial chemical composition and volatile evolution discussed in the following sections. Based on the time at which oxidation defects became perceptible according to IOC sensory criteria for the extra virgin olive oil category, the investigated cultivars were classified into two groups according to their sensory shelf life. A short shelf-life group included Frantoio (San Piero a Sieve), Gentile di Chieti, Itrana and Leccino, which developed sensory defects within 12 months after bottling, whereas the long shelf-life group comprised Frantoio (Siena) and Intosso, whose sensory quality remained compliant with the IOC standards for the extra virgin olive oil category for at least 12 months of storage. Collectively, these results demonstrate that, despite identical storage conditions, the investigated oils followed distinct sensory deterioration trajectories, indicating that the maintenance of the extra virgin category depends on intrinsic compositional characteristics rather than storage time alone.

### 2.3. VOC Analysis

**Figure 2:**
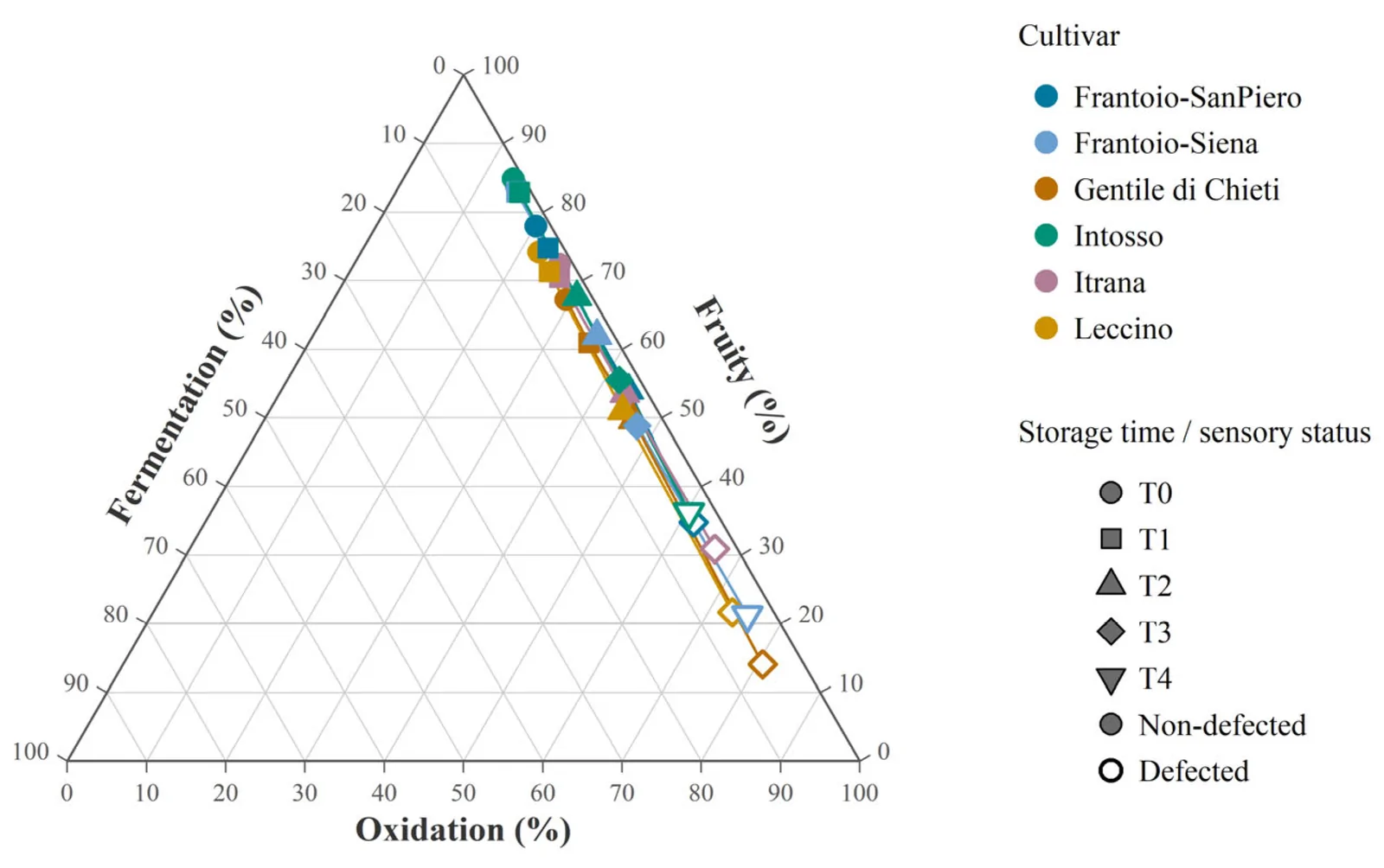
Ternary diagram showing the relative contribution of the three major classes of VOCs associated with olive oil aroma evolution during storage: fruity index (*m*/*z* 79.049, 81.069 and 99.080; lipoxygenase-derived compounds), oxidation index (*m*/*z* 45.033) and fermentation index (*m*/*z* 47.049, 59.049 and 61.028). For each sample and storage time, the summed signal intensity of each VOC class was normalized to the total intensity of the three classes so that their relative contributions summed to 1. Each point represents one olive oil sample at a given storage time (T0–T4), allowing visualization of the progressive shift from LOX-derived fruity volatiles toward oxidation- and, to a lesser extent, fermentation-related VOCs during storage. Colours identify the cultivars, symbol shapes indicate the storage times, and open symbols denote samples in which sensory defects were detected by the panel test.

In parallel with the sensory evaluation, the volatile fingerprint of each olive oil and its evolution during storage were monitored by PTR-ToF-MS. The following discussion focuses on VOC markers previously identified as the most informative indicators of extra virgin olive oil sensory quality [13,30]. Although all oils showed a broadly similar qualitative volatile profile, marked cultivar-dependent differences were observed in the emission intensity of these signals. These cultivar-specific volatile fingerprints were already evident at T0 and served as the starting point for the different storage trajectories described below. Based on their sensory relevance, the monitored VOCs were grouped into three categories. LOX-derived VOCs are among the main compounds responsible for the characteristic green and fruity aroma of high-quality virgin olive oils [30,31,32]. In the present study, this class was represented by the signals at *m*/*z* 79.049, 81.069, and 99.080, previously identified as LOX-derived VOCs by Taiti et al. [13]. The oxidation marker was represented by *m*/*z* 45.033, previously identified as one of the most reliable indicators of lipid oxidation and the rancid sensory defect [13]. Finally, fermentation-related VOCs, commonly associated with fermentative processes in virgin olive oils, were represented by the signals at *m*/*z* 47.049, 61.027, and 89.059, assigned to ethanol, acetic acid (or acetates), and butanoic acid, respectively, as reported by Taiti et al. [13]. These compounds have been consistently related to microbial activity and fermentative defects such as winey–vinegary and fusty–muddy sediment [33,34,35,36]. Cultivar-specific differences were already evident immediately after production (T0), particularly for the LOX-derived VOCs associated with positive sensory attributes. The summed emission of the three LOX-derived signals ranged from 232.62 ppbv in Gentile di Chieti to 576.11 ppbv in Intosso. Frantoio produced in Siena and Frantoio produced in San Piero a Sieve also exhibited comparatively high initial emissions (483.72 and 427.83 ppbv, respectively), whereas Itrana and Leccino showed intermediate values (348.75 and 268.91 ppbv). In all cultivars, *m*/*z* 81.069 was the predominant contributor to the LOX-derived fraction. These differences are consistent with the well-established influence of genotype, fruit maturity, growing conditions and processing technology on the formation of LOX-derived VOCs in virgin olive oil [31,37]. Moreover, they established markedly different initial pool of positive VOCs, which likely contributed to the cultivar-dependent evolution observed during storage. During the first four months of storage (T1), the volatile profile remained comparatively stable. The earliest detectable change was a moderate decline in the LOX-derived VOCs, which exceeded 9% in Frantoio (San Piero a Sieve) and 7% in Gentile di Chieti, whereas the remaining cultivars exhibited only minor reductions. In contrast, the oxidation marker (*m*/*z* 45.033) increased only slightly, while fermentation-related VOCs remained a minor component of the volatile profile (Table 3). This behaviour closely paralleled the sensory evaluation, as all oils remained free of detectable defects and continued to satisfy the IOC requirements for the EVOO category despite the slight reduction in fruitiness. Overall, these observations indicate that the earliest stage of storage was characterized primarily by the gradual depletion of LOX-derived VOCs, whereas the accumulation of oxidation- and fermentation-related compounds was still limited. The overall evolution of the volatile fingerprint is summarized in the ternary diagram (Figure 2), which integrates the relative contribution of the fruity, oxidation- and fermentation-related VOC classes into a single compositional representation. At T0, all oils clustered close to the fruity vertex, reflecting the predominance of LOX-derived VOCs over oxidation- and fermentation-related compounds. As storage progressed, the trajectories gradually shifted towards the oxidation domain, whereas the contribution of fermentation-related VOCs remained consistently limited throughout the experiment.

A more pronounced modification of the volatile fingerprint became evident between four and eight months of storage (T2). By this stage, the summed emission of LOX-derived VOCs had declined by approximately 19–32% relative to T0, although the extent of this reduction remained cultivar-dependent. Most of the decrease resulted from the progressive depletion of *m*/*z* 81.069 and 99.080, two major LOX-derived compounds generated during crushing and malaxation that are known to undergo oxidative degradation during storage [29]. Consequently, the contribution of green and fruity aroma notes progressively diminished before secondary oxidation products accumulated to concentrations sufficient to generate a perceptible rancid defect [33,37]. Concurrently, the oxidation marker increased sharply in all cultivars, reaching values 73–168% higher than those measured at T0. Frantoio (San Piero a Sieve) already exhibited the highest oxidation-related emission, whereas Gentile di Chieti and Leccino showed the lowest abundance of LOX-derived VOCs. Although fermentation-related VOCs also increased during this period, their accumulation remained modest and their absolute abundance was consistently much lower than both the LOX-derived VOCs and the oxidation marker. Most importantly, despite these substantial instrumental changes, none of the oils developed detectable sensory defects after eight months of storage. The simultaneous depletion of LOX-derived VOCs and accumulation of the oxidation marker therefore demonstrate that PTR-ToF-MS detected the progression of oxidative deterioration before oxidative deterioration became perceptible to the sensory panel. Instrumental changes in the volatile fingerprint should therefore not be interpreted as synonymous with immediate sensory downgrading, but rather as evidence of an ongoing deterioration process that precedes the appearance of recognizable sensory defects.

The volatile trajectories of the individual oils became markedly differentiated after 12 months of storage (T3), when four of the six monovarietal oils developed an oxidation-related sensory defect. This cultivar-dependent divergence is clearly visualized in the ternary diagram (Figure 2), where each monocultivar oil follows a distinct trajectory through the compositional space despite sharing the same overall direction of evolution from the fruity towards the oxidation domain. At this stage, the PTR-ToF-MS data closely mirrored the sensory evaluation while revealing that the dynamic volatile balance between the depletion of LOX-derived VOCs and the accumulation of oxidation-related VOCs evolved differently among cultivars. Gentile di Chieti and Leccino exhibited the most advanced stage of deterioration. In both oils, the LOX-derived VOCs were almost completely depleted, with the summed emission decreasing to 56.67 and 80.48 ppbv, respectively, while the oxidation marker increased to 325.39 ppbv in Gentile di Chieti and 313.74 ppbv in Leccino (Table 3). Their sensory downgrading therefore reflected the combined effect of an extensive loss of positive aroma compounds and the substantial accumulation of oxidation-related VOCs. Itrana followed a different deterioration pathway. Unlike Gentile di Chieti and Leccino, it retained a relatively abundant pool of LOX-derived VOCs at T3 (191.47 ppbv), comparable to that measured in Frantoio produced in Siena. Nevertheless, it exhibited by far the highest emission of the oxidation marker (411.11 ppbv), the highest value recorded among all oils during storage. The development of an oxidation-related sensory defect despite the persistence of an appreciable fraction of LOX-derived VOCs demonstrates that the maintenance of positive aroma compounds alone is insufficient to preserve the EVOO sensory classification once oxidation products accumulate to perceptible levels. Frantoio (San Piero a Sieve) showed a similar, although less pronounced, evolution, combining a marked depletion of LOX-derived VOCs with a substantial increase in *m*/*z* 45.033, ultimately leading to the same sensory outcome. Overall, these results indicate that the sensory evolution of EVOO is governed by the dynamic volatile balance between the progressive depletion of LOX-derived VOCs and the simultaneous accumulation of oxidation-related VOCs, rather than by the absolute abundance of either volatile fraction alone. This interpretation is further supported by the behaviour of Frantoio (Siena) and Intosso, the only oils that still fulfilled the EVOO requirements after 12 months of storage. Both cultivars retained substantially higher emissions of LOX-derived VOCs than the defective oils. Intosso was particularly noteworthy, as its summed emission at T3 (311.38 ppbv) remained considerably higher than the initial value measured in Gentile di Chieti. Its larger initial reserve of LOX-derived VOCs therefore allowed a recognizable fruity character to persist for a substantially longer period. Nevertheless, neither Frantoio (Siena) nor Intosso remained chemically stable. The oxidation marker increased from 86.34 to 218.84 ppbv and from 94.78 to 235.09 ppbv, respectively, demonstrating that oxidative processes progressed continuously despite the absence of detectable sensory defects. This apparent discrepancy reflects the different nature of the two analytical approaches. PTR-ToF-MS records quantitative changes in volatile abundance regardless of their sensory relevance, whereas human perception depends on compound-specific odour thresholds, interactions among odorants and masking effects exerted by the remaining volatile fraction [29,33]. Consequently, the progressive increase in *m*/*z* 45.033 observed in Frantoio (Siena) and Intosso should not be interpreted as evidence of an immediate sensory defect, but rather as an early indicator of oxidative deterioration that remained below the sensory recognition capability of the panel under the experimental conditions. Similar behaviour has previously been reported for virgin olive oils in which defect-associated ions became detectable before the corresponding sensory defects were recognized by trained assessors [34,38]. Conversely, oils may still retain appreciable emissions of LOX-derived VOCs while being downgraded once oxidation-related VOCs exceed sensory perception thresholds [13]. By 16 months of storage (T4), both Frantoio (Siena) and Intosso had also developed an oxidation-related sensory defect, confirming that all olive oils followed the same overall deterioration pathway. Regardless of cultivar, prolonged storage progressively reshaped the volatile fingerprint through two concurrent processes: the depletion of LOX-derived VOCs, responsible for the characteristic green and fruity aroma of virgin olive oil, and the accumulation of oxidation-related VOCs, ultimately leading to the loss of the EVOO category. Although the timing of these changes differed markedly among cultivars, the overall pattern of volatile evolution was remarkably consistent. In contrast, the fermentation-related VOCs followed a distinctly different evolution. Their abundance gradually increased during storage, but remained modest compared with the changes observed for the oxidation marker. Even after 12–16 months, they represented only a minor component of the overall volatile profile. This behaviour is also reflected in the ternary diagram (Figure 2), where the volatile trajectories remain consistently distant from the fermentation vertex throughout storage. The absence of fermentative defects is consistent with the excellent initial quality of the oils, which were produced from healthy olives, immediately filtered after extraction and characterized by very low free acidity and peroxide values (Table 1). Previous studies have shown that winey–vinegary and fusty–muddy sediment defects originate from microbial degradation of proteins and fermentable sugars contained in microdroplets of vegetation water suspended in unfiltered oils [39,40]. By removing most of these aqueous microdispersions immediately after extraction, filtration reduces both the water available for microbial activity and the supply of fermentable substrates, thereby making filtered oils considerably less prone to the formation of fermentation-related VOCs and their associated sensory defects. The behaviour observed in the present study is fully consistent with this mechanism, explaining both the limited accumulation of fermentation-related VOCs and the complete absence of fermentative defects throughout storage. Conversely, oxidative deterioration represents an unavoidable consequence of storage. Lipid autoxidation continues after bottling and cannot be completely prevented, even when oils are protected from light and stored under controlled temperature, because oxygen cannot be entirely excluded and unsaturated fatty acids remain inherently susceptible to oxidation [40,41]. The progressive increase in the oxidation marker observed in all cultivars therefore reflects the natural evolution of virgin olive oil during storage rather than inadequate processing or preservation conditions.

### 2.4. Chemical Determinants of Cultivar-Dependent Sensory Shelf Life

The previous sections demonstrated that, despite identical production standards and controlled storage conditions, the investigated monovarietal EVOOs followed distinct sensory and volatile deterioration trajectories during storage, resulting in substantial differences in the duration of their commercial extra virgin shelf life. This observation raised a key question: which initial compositional characteristics were associated with these different deterioration trajectories? To answer this question, we investigated whether the initial chemical composition of the oils (T0) could explain their subsequent shelf-life performance. Because all oils were produced according to recognized good practices for high-quality EVOO production and subsequently stored under identical conditions, differences in shelf-life behaviour were hypothesized to originate primarily from differences in their initial chemical composition rather than from technological or storage-related factors.

Three chemical parameters widely recognized as major determinants of the oxidative stability of virgin olive oils, namely the MUFA:PUFA ratio, total phenolic content and peroxide value, were selected as potential determinants of cultivar-dependent shelf life. The oils were grouped according to their observed storage performance: a long shelf-life group, comprising Frantoio (Siena) and Intosso, which remained free of sensory defects throughout the first 12 months of storage, and a short shelf-life group, including Frantoio (San Piero a Sieve), Gentile di Chieti, Itrana and Leccino, which developed oxidation defects by T3. Given the small and unbalanced number of independent oils in the two groups (*n* = 2 and *n* = 4), their initial compositional characteristics were compared descriptively and interpreted as exploratory (Table 4). The MUFA:PUFA ratio showed the clearest separation between groups, with higher values in the long-shelf-life oils, whereas total phenolic content and peroxide value showed less consistent differences. These observations indicate an association between the initial fatty acid balance and subsequent shelf-life behaviour, but do not provide definitive evidence of group-level differences. This finding is fully consistent with the well-established role of the MUFA:PUFA ratio as one of the principal determinants of the oxidative stability of virgin olive oils [16,42,43,44]. Because polyunsaturated fatty acids contain a higher number of double bonds than monounsaturated fatty acids, they are considerably more susceptible to lipid peroxidation. At a broader biological level, this susceptibility is consistent with the central role of reactive oxygen species in promoting oxidative damage to cellular components, including lipids, when their production exceeds antioxidant capacity under stress conditions [45]. Consequently, oils characterized by higher MUFA:PUFA ratios contain proportionally fewer highly oxidation-prone substrates and are expected to undergo oxidative deterioration more slowly, thereby preserving their sensory integrity for longer storage periods. Mechanistically, linoleic and linolenic acids represent the main polyunsaturated substrates of the LOX pathway leading to the formation of C5 and C6 aroma volatiles during olive oil extraction [46]. During storage, the lower susceptibility to oxidation associated with a higher MUFA:PUFA ratio may contribute to preserving the original LOX-derived volatile profile, thus providing a possible explanation for the slower decline observed in the more stable oils. The lack of a clear separation in total phenolic content between the two shelf-life groups should not binterpreted as evidence that phenolic compounds play only a minor role in EVOO stability. On the contrary, the antioxidant activity of phenolic compounds is well established, and numerous studies have demonstrated their ability to delay lipid oxidation and extend olive oil shelf life [7,47,48]. Therefore, our results indicate that phenolic concentration alone was insufficient to explain the observed cultivar-dependent differences in storage behaviour. For example, Frantoio produced in San Piero a Sieve exhibited the highest total phenolic concentration among all oils analysed, yet it developed oxidation defects after 12 months of storage. Conversely, Intosso maintained the EVOO category for the same storage period despite a lower phenolic concentration. Similarly, peroxide value showed little relationship with subsequent shelf life, as Frantoio produced in Siena combined the highest initial peroxide value with one of the longest sensory shelf lives, whereas Gentile di Chieti exhibited one of the lowest peroxide values despite belonging to the short shelf-life group. These observations indicate that neither peroxide value nor total phenolic content, when considered individually, adequately explained the cultivar-dependent differences in storage performance observed in the present study.

**Figure 3:**
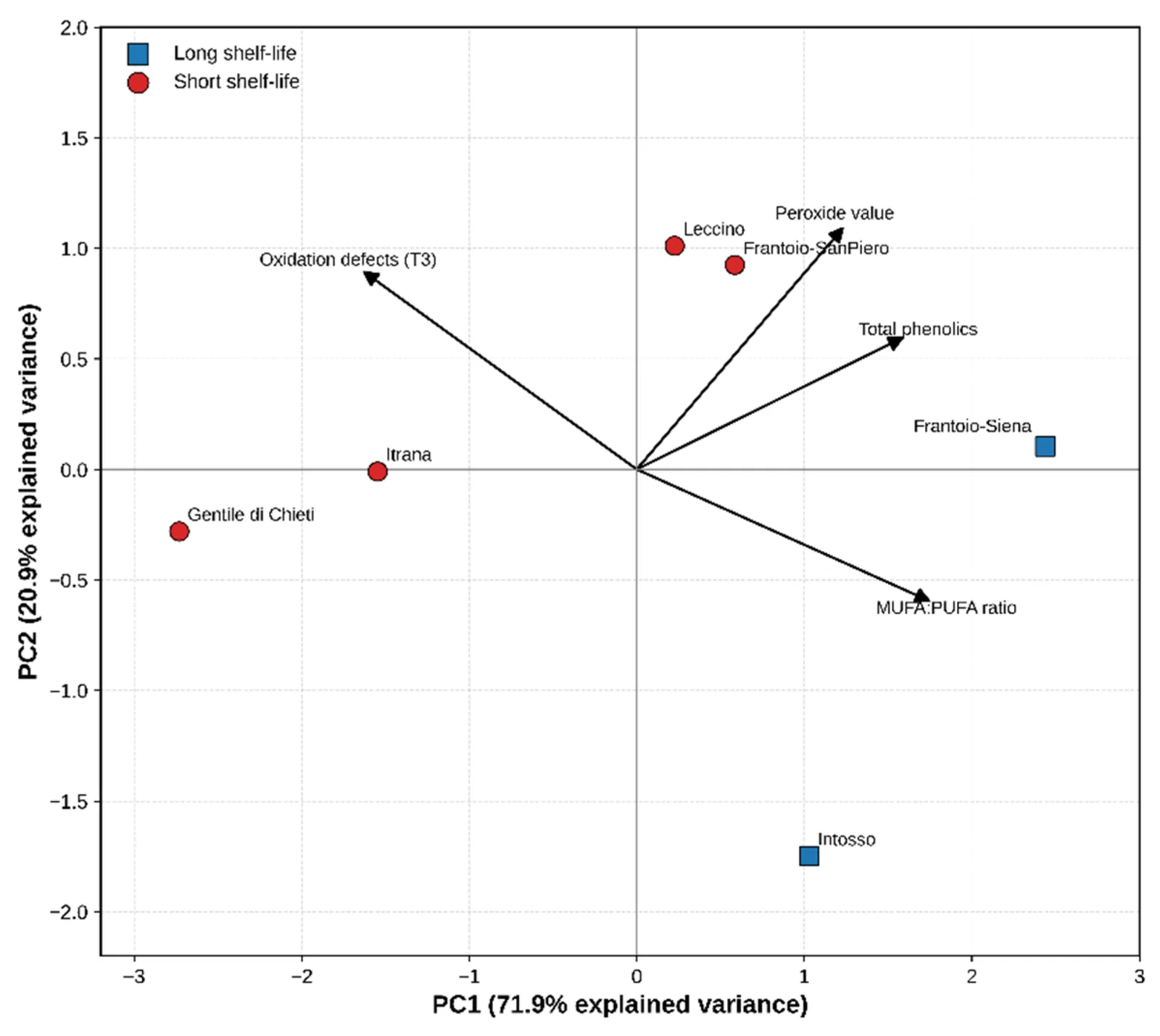
Principal component analysis (PCA) biplot of the investigated monovarietal extra virgin olive oils based on the initial peroxide value, total phenolic content, MUFA:PUFA ratio, and oxidation defect intensity after 12 months of storage (T3). Samples are colored according to their shelf-life classification (long vs. short shelf-life), and vectors indicate the contribution of each variable to the first two principal components, which explained 92.8% of the total variance.

In contrast, the MUFA:PUFA ratio consistently reflected the different deterioration trajectories observed throughout storage. Frantoio (Siena) and Intosso, which exhibited the highest MUFA:PUFA ratios, were the only oils that maintained the sensory requirements for classification as extra virgin olive oil throughout the first 12 months of storage. In contrast, Gentile di Chieti and Itrana, characterized by the lowest MUFA:PUFA ratios, not only lost the EVOO category by T3 but also exhibited the highest intensities of oxidation defects detected by the sensory panel. Frantoio (San Piero a Sieve) and Leccino, which displayed intermediate MUFA:PUFA ratios, also developed oxidation defects after 12 months of storage, although with lower defect intensities than Gentile di Chieti and Itrana. Collectively, these observations indicate that higher MUFA:PUFA ratios were associated not with the prevention of oxidative deterioration, but with a slower progression of the oxidative processes leading to the progressive depletion of LOX-derived VOCs, the accumulation of oxidation-related volatile compounds and, ultimately, the appearance of oxidation defects detected by the sensory panel. To explore the multivariate relationships among the investigated compositional variables and the observed shelf-life behaviour, PCA was performed as an exploratory analysis (Figure 3). Given the limited number of independent oils, the PCA was used only to visualize patterns and associations within the dataset and not for predictive purposes. The first two principal components explained 92.8% of the total variance, with PC1 accounting for 71.9% and PC2 for 20.9%. PC1 was positively associated with the MUFA:PUFA ratio (loading = 0.554), total phenolic content (0.502), and peroxide value (0.421), whereas oxidation defect intensity at T3 showed a negative loading (−0.514) (Table S1). Accordingly, Frantoio produced in Siena and Intosso, the two oils characterized by the longest sensory shelf life, were positioned on the positive side of PC1, whereas Gentile di Chieti and Itrana occupied the opposite side together with the highest oxidation-defect intensities. Frantoio (San Piero a Sieve) and Leccino assumed intermediate positions but remained associated with the short shelf-life group because both developed oxidation defects by T3. PC2 mainly separated Frantoio (Siena) from Intosso, reflecting the relatively greater contribution of peroxide value and phenolic content in the former and the higher MUFA:PUFA ratio in the latter.

Taken together, the descriptive comparison and exploratory PCA provide a coherent interpretation of the relationships between initial composition and the cultivar-dependent differences in shelf life observed in the present study. Among the investigated compositional variables, the MUFA:PUFA ratio emerged as the parameter most consistently associated with resistance to sensory deterioration during storage, whereas peroxide value and total phenolic content showed comparatively weaker individual relationships with storage performance. When interpreted together with the sensory evaluation and PTR-ToF-MS results, these findings indicate that oils characterized by higher MUFA:PUFA ratios possessed a lower intrinsic susceptibility to lipid oxidation, thereby slowing the progressive depletion of lipoxygenase-derived volatile compounds and delaying the accumulation of oxidation-related VOCs responsible for rancid sensory defects. Consequently, the investigated oils did not differ in the fundamental mechanism of oxidative deterioration, but rather in the rate at which this common deterioration trajectory progressed under identical storage conditions. This slower evolution of the volatile profile ultimately prolonged the maintenance of the sensory characteristics required for classification as extra virgin olive oil, resulting in a longer commercial shelf life. Although the limited and unbalanced number of oils does not allow robust inferential or predictive conclusions, the present results suggest that the initial MUFA:PUFA balance may be associated with the rate of sensory deterioration during storage and warrants validation in a larger set of monovarietal EVOOs.

## 3. Materials and Methods

### 3.1. Raw Material

Healthy olive fruits (*Olea europaea* L.) of the cultivars Frantoio, Leccino, Gentile di Chieti, Intosso, and Itrana, free from visible defects and signs of olive fruit fly infestation, were collected during the 2023/2024 harvest season from commercial orchards located in Tuscany and Abruzzo (Italy). The selected cultivars represented different genetic backgrounds and growing environments. Six experimental oils were obtained, including two Frantoio oils produced from olives grown in different areas (Siena and San Piero a Sieve, Florence), thus allowing the influence of growing environment within the same genotype to be evaluated. The orchards comprised both traditional and high-density intensive systems. Before milling, average fruit weight was determined and fruit ripening was assessed using the Jaén ripening index [49]. Detailed information on cultivar, geographical origin, altitude, orchard characteristics, fruit weight, Jaén ripening index, olive mill, and malaxation time is reported in Table 5. Overall, the selected olive batches encompassed a broad range of cultivars and growing environments, providing a suitable experimental framework for investigating factors associated with EVOO shelf life. For each experimental oil, olives were harvested on the day preceding milling using hand-held electric vibrating combs, collected on nets, and transferred into perforated plastic boxes containing approximately 20 kg of fruit. The olives were transported directly to the mill and stored overnight under shaded conditions in the same perforated boxes. They were processed the following day, within 24 h of harvest, using commercial continuous extraction systems operating under standard industrial conditions for high-quality EVOO production. Malaxation was carried out at 28 ± 1 °C for either 20 or 25 min, depending on the olive batch (Table 5). These conditions were selected to reflect common industrial practices, in which relatively short malaxation times (20–25 min) are commonly adopted to limit the loss of volatile compounds and preserve sensory quality [15].

### 3.2. Olive Oil Sample Collection

Immediately after extraction, 10 L of each of the six monovarietal EVOOs described in Section 3.1 were collected for the 16-month storage experiment. To minimize hydrolytic and fermentative degradation during storage and focus primarily on oxidative changes, the oils were immediately filtered using a plate filter press (Mori-TEM Srl, Florence, Italy) equipped with disposable cellulose filter sheets (CKP V8, Cordenons, Milan, Italy; nominal pore size 12 μm). Filtration was performed in a single pass at a flow rate of 28 L min^−1^. The removal of suspended solids and residual vegetation water through filtration reduces hydrolytic and fermentative processes and the development of associated sensory defects during storage [5,50]. The filtered oils were immediately bottled in 500 mL dark glass bottles, hermetically sealed, and stored at 15 ± 1 °C in a temperature-controlled chamber. A new bottle was opened at each sampling time for each analytical determination to avoid artefacts associated with repeated oxygen exposure. Sensory evaluation and volatile analyses were performed ten days after bottling (T0) and after 4 (T1), 8 (T2), 12 (T3), and 16 (T4) months of storage. Chemical quality parameters were determined only at T0, as the experimental design aimed to investigate whether the initial chemical composition of each oil was associated with its subsequent sensory and volatile evolution during storage.

### 3.3. Chemical Analysis

The fatty acid composition of each EVOO was determined according to the analytical methods described in Commission Regulation (EEC) No. 2568/91 and its subsequent amendments. Free acidity (FA), peroxide value (PV) and total phenolic content (TPC) were determined using an Olive Oxytester unit (CDR, Ginestra Fiorentina, Florence, Italy) according to the manufacturer’s instructions. Aliquots of 2.5, 5.0 and 10.0 μL were used for the determination of FA, PV and TPC, respectively. FA and PV rely on chromogenic reactions whose absorbance, read at 630 nm, is proportional to the concentration of free fatty acids and hydroperoxides, and are expressed as % oleic acid and meq O_2_ kg^−1^. TPC is based on a redox reaction in which the reducing compounds of the oil decolourise a chromogenic radical reagent in alcoholic solution; the decrease in absorbance at 505 nm is proportional to the phenolic concentration and is expressed as mg kg^−1^ tyrosol equivalents. Each EVOO was analysed in triplicate using three independently bottled samples to account for bottle-to-bottle variability and ensure analytical reliability. All chemical analyses were performed on oils collected at the first sampling time (T0).

### 3.4. VOC Analysis

The volatile fingerprint of each EVOO was monitored at four-month intervals during storage using proton-transfer-reaction time-of-flight mass spectrometry (PTR-ToF-MS). Analyses were performed only while the corresponding oils continued to fulfil the IOC sensory requirements for classification as extra virgin olive oil and were discontinued once sensory defects were detected. Analyses were performed using a PTR-ToF-MS instrument (Model 8000, Ionicon Analytik GmbH, Innsbruck, Austria) with H_3_O^+^ as the reagent ion over the mass range *m*/*z* 20–250. Sample preparation followed the protocol described by Taiti et al. [13]. Specifically, 15 g of oil was placed in a sealed 3/4 L glass jar (Bormioli) fitted with two Teflon septa, flushed with clean air for 60 s, incubated at 25 °C for 120 s to allow headspace equilibration, and then analyzed. A flow of 0.5 L min^−1^ of clean air was maintained at both the sampling device and the PTR-MS inlet. Between measurements, clean air was passed through the PTR-MS for 3 min to avoid memory effects. Mass spectra were recorded every second for 120 s per sample. The PTR-ToF-MS was operated at a drift-tube pressure of 2.20 mbar, drift temperature of 60 °C, and drift voltage of 600 V (E/N = 138 Townsend). All samples were analyzed in triplicate. For high mass resolution, spectra were recalibrated offline using *m*/*z* 21.022 (H_3_O^+^), 29.999 (NO^+^), and 59.049 (C_3_H_7_O^+^) as reference points [51]. The high mass resolution enabled the tentative assignment of VOCs on the basis of their accurate mass, and data were acquired and processed using TofDaq software v.183 (Tofwerk AG, Thun, Switzerland). Concentrations were expressed in ppbv following Lindinger and Jordan [52]. Only signals exceeding the 0.250 ppbv threshold were retained for subsequent analyses, whereas signals attributable to water chemistry or interfering ions were excluded prior to data processing. This analytical protocol was used to monitor the longitudinal evolution of the volatile fingerprint of each oil throughout the period during which it retained the sensory characteristics required for classification as extra virgin olive oil. For subsequent interpretation, PTR-ToF-MS signals were grouped into fruity (LOX-derived), oxidation-related and fermentation-related VOC classes according to the classification reported by Marone et al. [30] and Taiti et al. [13].

### 3.5. Sensory Analysis

All samples were subjected to sensory evaluation according to the official procedures of the International Olive Council [53,54,55] by a panel of eight assessors trained in accordance with IOC guidelines [56]. Positive sensory attributes (fruitiness, bitterness and pungency) and sensory defects were evaluated using the IOC profile sheet and a 10-cm intensity scale. According to IOC classification criteria, oils were classified as EVOO when the median of defects was equal to zero and the median of fruitiness was greater than zero. Sensory assessments were performed in a dedicated tasting room meeting the prescribed requirements, under adequately ventilated conditions and in the absence of extraneous odors [53,55]. Sensory evaluation was performed in parallel with PTR-ToF-MS analyses at each sampling time and represented the reference method for determining whether oils retained the extra virgin category throughout storage.

### 3.6. Data Analysis

To facilitate the interpretation of the volatile data, signals exceeding the 0.250 ppbv threshold were grouped into three functional VOC classes according to their established sensory significance: fruity (*m*/*z* 79.049, 81.069 and 99.080, corresponding to lipoxygenase (LOX)-derived VOCs), oxidation-related (*m*/*z* 45.033) and fermentation-related (*m*/*z* 47.049, 59.049 and 61.028) [13]. In particular, the *m*/*z* signals included in the fruity class showed strong positive correlations with pleasant sensory attributes of EVOO, including apple, ripe fruity, green tomato, grass and green notes [30]. For each sample and storage time, the emission intensities of the selected protonated masses were summed to generate three composite VOC indices describing the fruity, oxidation-related and fermentation-related volatile fractions. Unlike conventional chemometric indices based on standardized variables, these indices were calculated as the arithmetic sum of the original PTR-ToF-MS signal intensities, thereby preserving the quantitative relationships among the selected VOC markers while providing biologically meaningful descriptors of the volatile fingerprint. The relative contribution of each VOC class was then calculated by normalizing the three composite indices so that their sum equaled one. The normalized data were then visualized using a ternary diagram, allowing direct comparison of the balance between fruity, oxidation and fermentation-related volatile signatures among cultivars and throughout storage. The ternary representation emphasizes changes in the relative balance among the three VOC classes rather than their absolute emission intensities. Calculations and graphical representations related to the VOC composite indices and ternary diagram were performed in R (version 4.5.0; R Core Team, R Foundation for Statistical Computing, Vienna, Austria). All analytical determinations were performed on three biologically independent olive oil samples, and results are expressed as mean ± standard deviation. Differences among cultivars at the initial sampling time (T0) were evaluated by one-way analysis of variance (ANOVA), followed by Tukey’s honestly significant difference (HSD) test for multiple comparisons. Means sharing the same lowercase letter were not significantly different (*p* < 0.05). To identify the initial compositional characteristics associated with storage stability, olive oils were subsequently classified according to their shelf-life outcome. Oils that remained free of sensory defects after 12 months of storage (T3) were classified as long shelf-life, whereas oils exhibiting sensory defects at T3 were classified as short shelf-life. Given the small and unbalanced number of independent oils in the two shelf-life groups (*n* = 2 and *n* = 4), their initial compositional characteristics were compared descriptively and interpreted as exploratory. Principal component analysis (PCA) was performed as an exploratory multivariate approach to visualize relationships between initial chemical composition and subsequent shelf-life behaviour. Given the limited number of independent oils, PCA was not intended or used for predictive purposes. Variables were z-standardized prior to PCA. Statistical analyses were performed using XLSTAT software 2022 (Addinsoft, Paris, France).

## 4. Conclusions

The present study provides evidence that the sensory shelf life of extra virgin olive oil should not necessarily be regarded as a fixed period after bottling, but rather as the outcome of a progressive oxidative deterioration process whose rate may be influenced by the initial chemical composition of the oil. Although all investigated monovarietal EVOOs complied with IOC requirements at bottling and were produced and stored under comparable conditions, they exhibited markedly different rates of sensory and volatile evolution during storage. The integrated application of IOC sensory evaluation and PTR-ToF-MS monitoring showed that all oils followed the same fundamental oxidative deterioration pathway, characterized by the progressive depletion of lipoxygenase-derived volatile compounds and the concurrent accumulation of oxidation-related VOCs, ultimately leading to the appearance of oxidation defects. The principal difference among cultivars therefore lay not in the nature of the deterioration process itself, but in the rate at which this common trajectory progressed. Among the investigated compositional variables, the MUFA ratio emerged as the parameter most consistently associated with cultivar-dependent shelf life. Although oxidative stability results from the combined interaction between fatty acid composition and endogenous antioxidants, the present findings indicate that, under the experimental conditions adopted, the intrinsic susceptibility of the lipid fraction to oxidation was more consistently associated with the observed storage behaviour than either peroxide value or total phenolic content considered individually. Taken together, these findings suggest that a single best-before period may not adequately reflect the different deterioration rates of individual extra virgin olive oils. Rather than representing a fixed storage interval, commercial shelf life may depend on the rate at which each oil progresses along its oxidative deterioration trajectory, which may in turn be influenced by its initial chemical composition.

## Figures and Tables

**Table 1:** Quality parameters (free acidity, peroxide value and total phenolic content) and fatty acid composition of the extra virgin olive oil samples analysed in this study. Values are expressed as mean ± standard deviation (*n* = 3 biological replicates). Within each row, different lowercase letters indicate significant differences among cultivars according to Tukey’s HSD test (*p* < 0.05).

Parameter	Monovarietal EVOO					
	Frantoio-San Piero a Sieve	Frantoio-Siena	Gentile di Chieti	Intosso	Itrana	Leccino
Free acidity (%)	0.16 ± 0.01 ab	0.20 ± 0.02 a	0.13 ± 0.02 b	0.14 ± 0.02 b	0.13 ± 0.02 b	0.16 ± 0.01 ab
Peroxide value (meq O_2_ kg^−1^)	5.88 ± 0.03 b	7.00 ± 0.30 a	4.23 ± 0.23 c	4.27 ± 0.71 c	4.71 ± 0.34 c	6.00 ± 0.10 b
Total phenolic content (mg kg^−1^)	552.00 ± 15.00 a	501.50 ± 8.00 b	286.33 ± 16.11 d	475.15 ± 23.89 b	377.46 ± 12.78 c	508.00 ± 14.96 b
Oleic acid (%)	72.24 ± 0.45 c	73.11 ± 0.41 b	68.61 ± 0.18 e	74.36 ± 0.14 a	70.94 ± 0.18 d	71.53 ± 0.25 cd
Linoleic acid (%)	9.04 ± 0.07 c	7.32 ± 0.04 d	10.89 ± 0.10 a	7.54 ± 0.04 d	10.40 ± 0.23 b	8.88 ± 0.07 c
Linolenic acid (%)	0.84 ± 0.03 a	0.62 ± 0.04 b	0.65 ± 0.05 ab	0.77 ± 0.03 ab	0.62 ± 0.16 b	0.81 ± 0.02 ab
Palmitic acid (%)	13.44 ± 0.28 b	13.73 ± 0.17 b	14.86 ± 0.05 a	11.97 ± 0.07 c	13.54 ± 0.43 b	14.60 ± 0.24 a
Palmitoleic acid (%)	1.20 ± 0.04 a	1.16 ± 0.04 a	1.13 ± 0.06 a	0.90 ± 0.02 b	0.89 ± 0.03 b	1.18 ± 0.04 a
Stearic acid (%)	2.05 ± 0.04 c	2.58 ± 0.08 b	2.78 ± 0.10 b	3.26 ± 0.20 a	2.51 ± 0.08 b	2.14 ± 0.03 c
MUFA (%)	73.44 ± 0.49 c	74.27 ± 0.37 b	69.74 ± 0.14 e	75.26 ± 0.12 a	71.83 ± 0.15 d	72.71 ± 0.26 c
PUFAs (%)	9.88 ± 0.10 c	7.94 ± 0.03 f	11.54 ± 0.05 a	8.30 ± 0.03 e	11.02 ± 0.07 b	9.69 ± 0.05 d
MUFA:PUFA ratio	7.43 ± 0.02 c	9.36 ± 0.04 a	6.04 ± 0.04 e	9.06 ± 0.05 b	6.52 ± 0.05 d	7.50 ± 0.03 c

**Table 2:** Evolution of the sensory characteristics of the investigated olive oils during storage. The table reports the median of the predominant defect and fruitiness determined according to the IOC official panel test method at each sampling time. Bitterness and pungency were evaluated only at bottling (T0), since subsequent sensory assessments focused on the parameters required for the commercial classification of virgin olive oils according to IOC standards. Oils exhibiting oxidation as the predominant defect were no longer classified as extra virgin olive oils.

Sampling Times	Samples	Perceived Defects	Fruitiness	Commercial Category
T0	Frantoio-San Piero a Sieve	0	7	EVOO
	Frantoio-Siena	0	8	EVOO
	Gentile di Chieti	0	3	EVOO
	Intosso	0	8.5	EVOO
	Itrana	0	7.2	EVOO
	Leccino	0	5.4	EVOO
T1 (4 months)	Frantoio-San Piero a Sieve	0	6.6	EVOO
	Frantoio-Siena	0	7.8	EVOO
	Gentile di Chieti	0	3	EVOO
	Intosso	0	8.5	EVOO
	Itrana	0	7	EVOO
	Leccino	0	5.4	EVOO
T2 (8 months)	Frantoio-San Piero a Sieve	0	5.5	EVOO
	Frantoio-Siena	0	6.4	EVOO
	Gentile di Chieti	0	1.8	EVOO
	Intosso	0	6.8	EVOO
	Itrana	0	6	EVOO
	Leccino	0	5.2	EVOO
T3 (12 months)	Frantoio-San Piero a Sieve	1.2 (Oxidation)	3.2	Not EVOO
	Frantoio-Siena	0	4	EVOO
	Gentile di Chieti	2.0 (Oxidation)	1.1	Not EVOO
	Intosso	0	4.2	EVOO
	Itrana	1.6 (Oxidation)	3.3	Not EVOO
	Leccino	1.4 (Oxidation)	2	Not EVOO
T4 (16 months)	Frantoio-Siena	1.2 (Oxidation)	3	Not EVOO
	Intosso	1.0 (Oxidation)	3.5	Not EVOO

**Table 3:** Mean emission intensities (ppbv ± SD) of the selected PTR-ToF-MS volatile markers monitored during storage of six monovarietal extra virgin olive oils. The protonated masses *m*/*z* 79.049, 81.069 and 99.080 represent lipoxygenase (LOX)-derived VOCs associated with positive fruity aroma; *m*/*z* 45.033 was used as an oxidation marker, whereas *m*/*z* 47.049, 61.027 and 89.059 represent fermentation-related VOCs. The last three columns report the corresponding Fruity, Fermentation and Oxidation indices, calculated as the arithmetic sum of the selected marker ions belonging to each VOC class. Oils were classified as EVOO and not EVOO according to the IOC sensory evaluation. ND = not detected or below the 0.250 ppbv threshold.

Sampling Times	Samples	Panel Evaluation (EVOO/non EVOO)	*m*/*z* 45.033	*m*/*z* 47.049	*m*/*z* 61.027	*m*/*z* 79.049	*m*/*z* 81.069	*m*/*z* 89.059	*m*/*z* 99.080	Fruity Index	Fermentation Index	Oxidation Index
T0	Frantoio-San Piero a Sieve	EVOO	111.32 ± 6.18	0.40 ± 0.10	10.24 ± 1.36	3.44 ± 0.25	403.30 ± 26.85	ND	28.09 ± 2.61	434.83	10.64	111.32
	Frantoio-Siena	EVOO	86.34 ± 11.82	0.35 ± 0.10	8.25 ± 1.53	3.60 ± 0.33	451.89 ± 29.39	ND	30.23 ± 1.93	485.72	8.60	86.34
	Gentile di Chieti	EVOO	102.00 ± 8.13	0.32 ± 0.11	11.86 ± 1.61	1.50 ± 0.41	216.25 ± 13.80	ND	15.87 ± 1.65	233.62	12.18	102.00
	Intosso	EVOO	94.78 ± 12.16	0.37 ± 0.14	8.75 ± 1.62	2.41 ± 0.22	536.90 ± 31.61	ND	38.80 ± 2.68	578.11	9.12	94.78
	Itrana	EVOO	120.81 ± 14.17	0.43 ± 0.13	8.34 ± 1.57	4.76 ± 0.65	321.41 ± 20.86	ND	26.58 ± 2.86	352.75	8.77	120.81
	Leccino	EVOO	90.54 ± 11.76	0.40 ± 0.11	11.44 ± 1.14	1.90 ± 0.72	281.60 ± 27.70	ND	20.61 ± 1.82	304.11	11.84	90.54
T1 (4 months)	Frantoio-San Piero a Sieve	EVOO	124.94 ± 5.04	0.38 ± 0.11	10.24 ± 1.40	3.39 ± 0.54	375.04 ± 18.80	ND	22.59 ± 1.32	401.02	10.62	124.94
	Frantoio-Siena	EVOO	87.00 ± 12.17	0.34 ± 0.11	10.25 ± 1.05	3.42 ± 0.73	445.00 ± 20.38	ND	25.30 ± 3.98	473.72	10.59	87.00
	Gentile di Chieti	EVOO	120.30 ± 15.66	0.41 ± 0.12	12.86 ± 1.42	1.15 ± 0.31	201.25 ± 17.87	ND	13.70 ± 1.56	216.10	13.27	120.30
	Intosso	EVOO	104.00 ± 8.62	0.33 ± 0.11	9.87 ± 1.38	2.33 ± 0.41	514.10 ± 13.73	ND	34.80 ± 3.50	551.23	10.20	104.00
	Itrana	EVOO	121.98 ± 20.72	0.46 ± 0.23	12.37 ± 1.20	3.55 ± 0.85	310.61 ± 10.22	ND	23.42 ± 1.72	337.58	12.83	121.98
	Leccino	EVOO	94.43 ± 8.27	0.42 ± 0.14	13.14 ± 2.36	1.70 ± 0.42	247.05 ± 18.77	ND	18.61 ± 2.76	267.36	13.56	94.43
T2 (8 months)	Frantoio-San Piero a Sieve	EVOO	209.44 ± 19.38	0.48 ± 0.21	11.89 ± 1.29	2.11 ± 0.34	288.87 ± 23.90	ND	19.26 ± 3.34	310.24	12.37	209.44
	Frantoio-Siena	EVOO	201.11 ± 14.48	0.34 ± 0.14	11.82 ± 2.32	2.20 ± 0.53	329.50 ± 10.08	ND	20.36 ± 2.64	352.06	12.16	201.11
	Gentile di Chieti	EVOO	176.78 ± 14.40	0.54 ± 0.12	13.73 ± 2.24	0.57 ± 0.22	181.41 ± 8.31	ND	6.70 ± 1.26	188.68	14.27	176.78
	Intosso	EVOO	196.82 ± 25.96	0.47 ± 0.27	11.77 ± 1.46	1.48 ± 0.35	415.55 ± 14.01	ND	19.18 ± 2.30	436.21	12.24	196.82
	Itrana	EVOO	218.29 ± 12.93	0.92 ± 0.23	13.13 ± 2.35	2.35 ± 0.61	252.26 ± 21.96	0.29 ± 0.11	13.66 ± 1.42	268.27	14.34	218.29
	Leccino	EVOO	170.54 ± 25.25	0.99 ± 0.33	13.64 ± 2.46	0.93 ± 0.21	192.44 ± 12.55	0.26 ± 0.05	10.55 ± 2.23	203.92	14.89	170.54
T3 (12 months)	Frantoio-San Piero a Sieve	non-EVOO	332.04 ± 12.12	0.86 ± 0.32	18.24 ± 3.64	2.41 ± 0.48	181.87 ± 13.07	0.41 ± 0.18	5.99 ± 1.19	190.27	19.51	332.04
	Frantoio-Siena	EVOO	218.84 ± 4.62	1.04 ± 0.36	15.82 ± 3.28	2.32 ± 0.64	209.50 ± 16.89	0.36 ± 0.11	13.11 ± 1.40	224.93	17.22	218.84
	Gentile di Chieti	non-EVOO	325.39 ± 6.08	1.91 ± 0.57	18.32 ± 2.39	ND	56.00 ± 8.56	0.65 ± 0.20	1.67 ± 0.42	57.67	20.88	325.39
	Intosso	EVOO	235.09 ± 8.98	0.52 ± 0.11	13.81 ± 1.34	1.53 ± 0.22	303.99 ± 14.51	0.40 ± 0.19	5.86 ± 0.77	311.38	14.73	235.09
	Itrana	non-EVOO	411.11 ± 10.24	1.50 ± 0.45	15.80 ± 2.26	1.10 ± 0.30	189.01 ± 8.66	0.57 ± 0.20	2.36 ± 0.55	192.47	17.87	411.11
	Leccino	non-EVOO	313.74 ± 9.51	1.73 ± 0.31	18.47 ± 2.89	0.35 ± 0.18	90.48 ± 11.46	0.52 ± 0.16	3.87 ± 0.83	94.70	20.72	313.74
T4 (16 months)	Frantoio-Siena	non-EVOO	372.04 ± 19.82	1.77 ± 0.42	17.62 ± 2.56	ND	109.80 ± 11.61	0.63 ± 0.25	0.70 ± 0.22	110.50	20.02	372.04
	Intosso	non-EVOO	308.34 ± 24.13	1.62 ± 0.35	16.81 ± 1.95	0.31 ± 0.05	183.99 ± 6.53	0.69 ± 0.34	0.86 ± 0.23	185.16	19.22	308.34

**Table 4:** Initial values (mean ± SD) of candidate indicators of oxidative stability in the long- and short-shelf-life groups. Values are presented descriptively because of the small and unbalanced number of independent oils in the two groups (*n* = 2 and *n* = 4, respectively).

Parameter	Long Shelf Life	Short Shelf Life
MUFA:PUFA ratio	9.21 ± 0.17	6.87 ± 0.65
Total phenolics (mg kg^−1^)	488.3 ± 21.5	431.0 ± 110.7
Peroxide value (meq O_2_ kg^−1^)	5.64 ± 1.57	5.21 ± 0.81

**Table 5:** Characteristics of the olive batches used for extra virgin olive oil production, including cultivar, geographical origin (region and district), altitude of the olive-growing area, orchard type, fruit weight, Jaén ripening index at harvest, olive mill where processing was performed, and malaxation time.

Sample Number	Cultivar	Region	District	Altitude (m a.s.l.)	Orchard Type	Harvest Date	Harvest Type	Fruit Weight (g)	Jaén Ripening Index	Malaxation Time (min)
1	Frantoio	Tuscany	Siena	350	Traditional, ~300–350 trees/ha (20–30 yr orchard)	20 October	Hand-held olive harvesting	2.1 ± 0.2	3.0	25
2	Frantoio	Tuscany	San Piero a Sieve (Fi)	500	Traditional, ~300–350 trees/ha (30–40 yr orchard)	20 October	Hand-held olive harvesting	1.9 ± 0.3	2.8	25
3	Leccino	Tuscany	San Piero a Sieve (Fi)	500	High-density intensive, ~1000 trees/ha (10–12 yr orchard)	12 October	Self-propelled olive harvester	2.4 ± 0.3	3.1	20
4	Gentile di Chieti	Abruzzo	Chieti	300	Traditional, ~300–350 trees/ha (15–20 yr orchard)	24 October	Hand-held olive harvesting	2.8 ± 0.2	3.2	20
5	Intosso	Abruzzo	Chieti	300	Traditional, ~300–350 trees/ha (15–20 yr orchard)	24 October	Hand-held olive harvesting	3.3 ± 0.3	2.5	25
6	Itrana	Abruzzo	Chieti	300	Traditional, ~300–350 trees/ha (15–20 yr orchard)	24 October	Hand-held olive harvesting	4.5 ± 0.3	1.4	25

## Data Availability

Data are contained within the article and Supplementary Materials.

## Supplementary Materials

The following supporting information can be downloaded at https://www.mdpi.com/article/10.3390/plants15182834/s1, Table S1: PCA loadings of the variables included in the analysis. PCA was performed on z-standardized variables. Loadings represent the coefficients of the original standardized variables on each principal component.

## Abbreviations

The following abbreviations are used in this manuscript:

EVOO	Extra Virgin olive oil
VOCs	Volatile Organic Compounds
