# Supplementary material for: From Composition to Shelf Life: Oxidative Deterioration Trajectories of Monovarietal Extra Virgin Olive Oils
Source: Plants (Basel). 2026 Sep 16;15(18):2834. doi: 10.3390/plants15182834 (PMC13610343; doi:10.3390/plants15182834)
Supplement: Supplementary file 1 [file plants-15-02834-s001.zip › plants-4531163-supplementary.pdf]

Supplementary Materials

Table S1. PCA loadings of the variables included in the analysis. PCA was performed on z-standardized variables. Loadings represent the coefficients of the original standardized variables on each principal component.

**Variable**	**PC1**	**PC2**
Peroxide value	0.421	0.688
Total phenolic content	0.502	0.354
MUFA:PUFA ratio	0.554	-0.357
Oxidation defect intensity at T3	-0.514	0.524
**Explained variance (%)**	**71.9**	**20.9**
**Cumulative variance (%)**	**71.9**	**92.8**
